# Sp17 gene expression in myeloma cells is regulated by promoter methylation

**DOI:** 10.1038/sj.bjc.6602160

**Published:** 2004-09-21

**Authors:** Z Wang, Y Zhang, B Ramsahoye, D Bowen, S H Lim

**Affiliations:** 1 Division of Hematology and Oncology, Texas Tech University Health Sciences Center, Amarillo, TX, USA; 2 Biotherapy and Stem Cell Transplant Program, Don and Sybil Harrington Cancer Center, 1500 Wallace Boulevard, Amarillo, TX 79106, USA

**Keywords:** sperm protein 17, gene expression, promoter methylation

## Abstract

The mechanisms underlying sperm protein 17 (Sp17) gene expression in myeloma cells remained unclear. Using reverse transcription–polymerase chain reaction (RT–PCR), Sp17 transcripts were detected in ARK-B, ARP-1, RPMI-8226 and KMS-11 but not in H929, IM-9, MM1-R and U266 cells. Using a panel of primer pairs in methylation-sensitive PCR to amplify overlapping gene segments, our screening studies showed that the *Hpa*II sites at −359 and −350 are involved in the regulation of Sp17 gene expression. To confirm the differences in methylation status between Sp17-positive and Sp17-negative cell lines, KMS-11 cells (Sp17-positive) and IM-9 cells (Sp17-negative) were subjected to the more accurate method of bisulphite conversion. KMS-11 cells were more hypomethylated at these *Hpa*II sites of exon 1 compared to IM-9 cells, indicating the association of hypomethylated promoter with Sp17 gene expression. In addition, the level of methylation at other CpG sites within the promoter sequence was also higher in IM-9 than KMS-11. Exon 1 was cloned into a reporter vector, pCAT^*^3 Enhancer. Chloramphenicol acetyl transferase (CAT) activity was restored in cells transfected with the recombinant plasmid, indicating the promoter function of exon 1. Exposure of Sp17-negative cell lines to the hypomethylating agent, 5-azacytidine, resulted in the upregulation of Sp17 gene expression. Our results therefore provide evidence for the regulation of Sp17 gene expression by promoter methylation.

Sperm protein 17 (Sp17) is a 22–24 kDa normal testicular protein aberrantly expressed by tumour cells from patients with multiple myeloma (MM) (Lim *et al*, 2001) and ovarian cancer (Chiriva-Internati *et al*, 2002a). It has also been found to be expressed by tumour cells from some malignant melanoma, astrocytoma and colon cancer (unpublished). It is a cancer-testis (CT) antigen expressed only in normal testis and some tumour cells but not in any other normal tissues (Lim *et al*, 2001; De Jong *et al*, 2002). Sperm protein 17-specific CD8+ cytotoxic T-lymphocytes (CTLs) reactive with Sp17-positive HLA-matched fresh tumour cells could be generated from the peripheral blood of healthy donors (Chiriva-Internati *et al*, 2001, 2002b) and cancer-bearing patients (Chiriva-Internati *et al*, 2002a, 2002c). The restricted normal tissue expression of Sp17 and the ability to generate tumour-reactive specific CTLs suggest that Sp17 may be a potential target for tumour vaccines of Sp17-positive tumours.

Sp17 is involved in the interaction of spermatozoa with oocytes after acrosome reaction (in mouse) (Kong *et al*, 1995) and after sperm–zona pellucida contact is initiated (in rabbit) (Richardson *et al*, 1994). In addition, it has a functional role in cell regulation by participating in signalling pathways through its calmodulin binding site at the C-terminal (Wen *et al*, 2001). The N-terminal is similar to a cAMP-dependent protein kinase regulatory subunit and the central domain participates in heparin binding (Lacy and Sanderson, 2001; Wen *et al*, 2001). The gene encoding Sp17 is mapped by sequence database to chromosome 11 in human. It contains five exons and four introns (Buchli *et al*, 2002). There are two transcriptional start sites, designated Sp17-1a and Sp17-1b. Recently, an intronless Sp17 pseudogene (Sp17-2) has been identified in human in chromosome 10 and is attributed to RNA retroposition.

COS cells transfected with Sp17 cDNA showed Sp17 in their cytoplasm (Yamasaki *et al*, 1995). Although Sp17 does not have a transmembrane domain, Sp17 was also found on the surface of live COS cells. The explanation for this observation is not apparent. It is possible that Sp17 has an unknown mechanism for arriving at the cell surface by lipid associations or carrier proteins. Alternatively, it could have arisen due to cell death and subsequent release of cytoplasmic Sp17 that became associated with the membrane.

Even though Sp17 shows a restricted normal tissue expression, the mechanisms regulating Sp17 aberrant expression in myeloma cells are not known. We therefore set out to determine the mechanisms of the Sp17 gene expression since such information may provide the opportunity to manipulate the gene for therapeutic purposes. Changes in gene methylation occur frequently in cells that have undergone malignant transformation. These changes are thought to be responsible for the aberrant expression of the genes encoding some CT antigens. Since Sp17 is a CT antigen, we hypothesised that Sp17 gene expression might be controlled by methylation of the promoter sequence so that the gene is silenced by DNA methylation. To test this hypothesis, we have investigated Sp17 gene expression in eight MM cell lines and compare the effects and status of promoter methylation to Sp17 status of the tumour cell lines.

## MATERIALS AND METHODS

### MM cell lines

Eight MM cell lines were used: ARK-B and ARP-1 (gifts from Joshua Epstein, PhD, University of Arkansas for Medical Sciences, Little Rock, AR), H929, KMS-11 and RPMI 8226 (gifts from Raymond Comenzo, MD, Memorial Sloan Kettering Cancer Center, New York, NY), IM-9 and U266 (gifts from Dharminder Chanhan, PhD, Dana Farber Cancer Center, Boston, MA) and MM1-R (gift from Steve Rosen, MD, Northwestern University, Chicago, IL). All cell lines were maintained at liquid culture prior to being used for the experiments.

### Reverse transcription–polymerase chain reaction (RT–PCR) amplification of Sp17 mRNA

Reverse transcription–polymerase chain reaction performed. Briefly, all RNA specimens were first treated with DNAse I (Ambion Inc., Austin, TX, USA) to remove genomic DNA contamination. First-strand cDNA was synthesised from 1 *μ*g of total RNA using a random hexamer primer. The PCR primers were 5′SP17PCR: 5′-CAG CAG AAT GGG GGA GTA AG-3′; and 3′SP17PCR: 5′-CAG CTT GGA TTT TGA CAG CA-3′. Polymerase chain reaction was performed using 35 amplification cycles at an annealing temperature of 60°C. Negative controls in all the PCR reactions contained the PCR reaction mixture except for cDNA, which was substituted with water. RNA integrity in each sample was checked by amplification of a *β*-actin gene segment. Successful removal of genomic DNA contamination was confirmed in each sample by amplification of the RNA without prior RT reaction. Polymerase chain reaction products were visualised on an ethidium bromide agarose gel. All results were confirmed on two independent RT–PCRs.

### Methylation-sensitive PCR

A panel of oligonucleotide primers was used to amplify overlapping gene segments on the Sp17 exon 1 or a control gene segment in exon 5 of Sp17 gene (Table 1

**Table 1:** Oligonucleotide primers used for methylation-sensitive PCR analysis

**Primer**	**Primer sequences**	**Gene segment amplified *Hpa*II sites**	**Annealing temperature (°C)**
I	5′-CAC CCT TTT GCA GTC CTC GCA GC-3′	From position −186 to −28	60
	5′-CTC CTT TCT CTC CGA GCT GGT GC-3′	(IA and IB)	
			
II	5′-CGC GAC CAT GCT TGC AAG GA-3′	From position –280 to –62	52
	5′-TTG TTT TTC TAG TTG CTG GG-3′	(II)	
			
III	5′-GCC GCC TCA CCT GCA CTT CTG TC-3′	From position –348 to –227	60
	5′-GAA CCC ACT TTC CCA GTC CTA GG-3′	(III)	
			
IV	5′-GCC TCG GTC GGA GAC GCG AGC CC-3′	From position –488 to –285	60
	5′-GGC TTG TAC TCG GGC TGG TGC TG-3′	(IVA and IVB)	
			
V	5′-GGT TCA ACC CAG CAG ACT CCT GC-3′	From position –518 to –362	60
	5′-ACC CCT GCC CCT CAG CCT GTG CG-3′	(V)	
			
VI	5′-ACC TAA CTA GCT TCG CCT CCT TC-3′	From position –693 to –493	60
	5′-TCT GCA GGA GTC TGC TGG GTT GA-3′	(VI)	
			
Control	5′-GGG ACA CAT AGC CAg AGA G-3′	Exon 5, position 377 to 559	52
	5′-TTC ACA ACA GTA ACG AAT G-3′		

PCR=polymerase chain reaction.

). Genomic DNA was extracted from tumour cells using a commercially available DNA extraction kit. The DNA was digested for 3 h at 37°C with 10 U *μ*g^−1^ methylation-sensitive restriction enzyme *Hpa*II, extracted with phenol and chloroform–isoamyl alcohol (25 : 24 : 1), and recovered by ethanol precipitation. To ensure completion of digestion, the above process was repeated. This restriction digest protocol has been used successfully in previous works (De Smet *et al*, 1996; Sigalotti *et al*, 2002) and has been found to result consistently in complete digestion of the genomic DNA by *Hpa*II restriction enzyme. Following ethanol precipitation, 100 ng of the digested DNA was used for amplification in a final volume of 50 *μ*l PCR mixture. Polymerase chain reaction was carried out for 30 cycles in a thermal cycler. Polymerase chain reaction products were analysed on a 2% agarose gel and visualised by ethidium bromide staining. All results were confirmed in two independent PCR.

### Sodium bisulphite genomic DNA modification

Genomic DNA was first digested with *Eco*RI, then denatured with 0.3 M NaOH for 15 min. The denatured DNA was then reacted with 3.6 M sodium bisulphite and 1 mM hydroquinone (55°C for 14 h). The DNA was desalted using a DNA clean-up kit (Wizard DNA Clean Up, Promega, Madison, WI) and precipitated for PCR. Polymerase chain reaction was carried out using the oligonucleotide primers that amplify the whole exon 1 of Sp17 gene: 5′-ACC TAA CTA GCT TCG CCT CCT TC-3′; and 5′-CTC CTT TCT CTC CGA GCT GGT GC-3′. The PCR products were cloned into the TA-cloning system. Eight recombinant clones were randomly picked from each transfection for nucleotide sequence analysis to determine the proportion of hypomethylated promoter sequence.

### *In vitro* methylation of plasmids, cell transfection and analysis of chloramphenicol acetyl transferase (CAT) expression

Sp17 promoter sequence contained within exon 1 was isolated, amplified and cloned into TA-cloning system for sequence analysis. The sequence was identical to that published recently (Buchli *et al*, 2002). We then subcloned the promoter DNA into the pCAT^*^3 Enhancer vector between *Kpn*I and *Bgl*II.

The plasmid was methylated in each CpG dinucleotide with *Sss*I methylase according to the manufacturer's instruction. Control unmethylated plasmid was obtained under similar experimental conditions but without the addition of the *Sss*I methylase. Methylated (pCAT^*^3 Enhancer-Sp17promoter/met) or unmethylated (pCAT^*^3 Enhancer-Sp17 promoter) plasmids were purified by phenol and chloroform extractions, followed by ethanol precipitation. Successful methylation of the plasmid was confirmed by restriction digest using methylation-sensitive *Hpa*II. Once generated, both pCAT^*^3 Enhancer-Sp17promoter/met and pCAT^*^3 Enhancer-Sp17promoter were used to transfect the human embryonic kidney cell line, 293 cells.

Transfection was carried out using the FuGENE 6 reagent (Roche Molecular Biochemicals) according to the manufacturer's recommendation. Briefly, the cells were seeded into a six-well cluster plate and grown to 50% confluence. The cultures were transfected with 2 *μ*g of the plasmids and assayed for CAT activities after 72 h. FAST CAT Green (deoxyl) Chloramphenicol Acetyltransferase Assay Kit (Molecular Probes, Eugene, OR, USA) was used to detect CAT activity. The transfectants were lysed and a cytoplasmic extract prepared. The extract was then incubated with the fluorescent deoxylchloramphenicol substrate and acetyl CoA at 37°C. The reaction was terminated by the addition of ice-cold ethyl acetate. After drying and dissolution in ethyl acetate, the reaction substrate and product were resolved by thin-layer chromatography on silica gel plates and eluted with a chloroform:methanol mixture (85 : 15 (v v^−1^)).

### *In vitro* treatment of myeloma cell lines with 5-azacytidine

Tumour cells were treated with 2 *μ*M 5-azacytidine. The medium was replaced daily with fresh culture medium and after 96 h, the cells were used for molecular analyses. Control cultures were grown under identical conditions but without 5-azacytidine.

### Northern blot analysis

Total RNA was prepared from the cells using a commercially available RNA preparation kit, RNAEasy (Qiagen, USA). Total RNA (10 *μ*g) from each cell preparation were electrophoresed on a 1.2% agarose/formaldehyde gel and transferred onto a nylon membrane (MSI, Westboro, MA, USA). Membranes were prehybridised in a commercial NorthernMax™ Prehyb/Hyb buffer (Ambion, Austin, TX, USA) for 1 to 3 h and then hybridised for 18 h at 42°C. Sperm protein 17 full-length cDNA (approximately 500 base pairs (bp)) and a control cDNA, *β*-actin fragment (615 bp), templates were labelled by random priming with [^32^P]dCTP using the Ready-to-Go DNA labelling kit (Amersham Pharmacia Biotech Inc., Pharmacia, Piscataway, NJ, USA). Final washes of the membranes were performed at 68°C with 1 × SSC with 0.25% sodium dodecyl sulfate (SDS) solution. Binding to Sp17 or *β*-actin mRNA was visualised by autoradiograph.

### Statistical analysis

Fisher's exact tests were used to determine the significance of differences between two variables. A *P*-value of ⩽0.05 was considered statistically significant.

## RESULTS

### Differential Sp17 gene expression in MM cell lines

**Figure 1:**
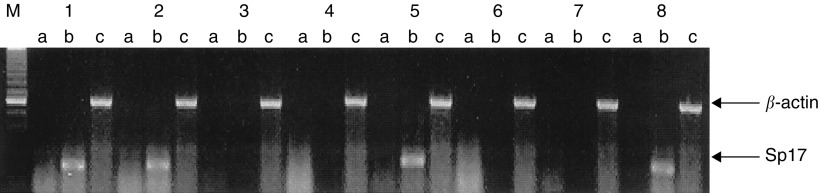
Reverse transcription–polymerase chain reaction analysis using a pair of sequence-specific primers for Sp17 produced a positive signal of approximately 500 bp (lane 1=ARK-B; lane 2=ARP-1; lane 3=H929; lane 4=IM-9; lane 5=KMS-11; lane 6=MM1-R; lane 7=U266; and lane 8=RPMI 8226) (M=molecular marker; lane a=PCR without prior RT; lane b=RT–PCR for Sp17; lane c=control amplification for a *β*-actin gene segment).

Using a pair of sequence-specific primers in PCR, we first determined the expression of Sp17 gene in eight MM cell lines. Sperm protein 17 mRNA was only detected in ARK-B, ARP-1, KMS-11 and RPMI 8226 and not the other four MM cell lines (Figure 1

). Although the PCR was not designed primarily to provide an accurate mRNA quantitation, the reproducibly more intense signal from KMS-11 suggests a higher level of Sp17 gene expression in KMS-11 when compared to ARP-1, ARK-B and RPMI 8226.

### Association between Sp17 gene expression and hypomethylation of specific *Hpa*II sites by methylation-sensitive PCR

**Figure 2:**
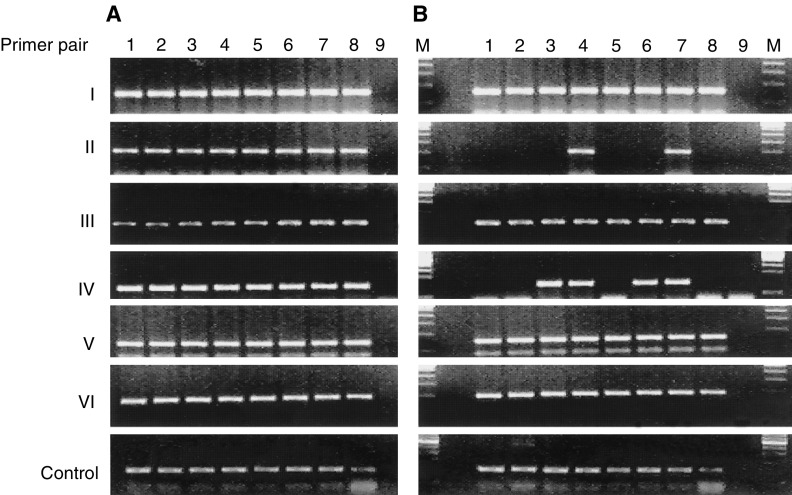
Polymerase chain reaction analysis for Sp17 promoter sequence using six primer pairs to amplify across eight *Hpa*II sites. Therefore, PCR products were only obtained if the target sequence are methylated at the *Hpa*II sites (**A**=PCR without prior *Hpa*II digest; **B**=PCR after *Hpa*II digest) (lane 1=ARK-B; lane 2=ARP-1; lane 3=H929; lane 4=IM-9; lane 5=KMS-11; lane 6=MM1-R; lane 7=U266; and lane 8=RPMI 8226) (M=molecular marker).

To determine if the heterogeneity of Sp17 gene expression among the MM cell lines was related to differences in the methylation status of their promoters, we first used *Hpa*II restriction digest/PCR analysis to screen the Sp17 promoters in these cell lines. A total of eight *Hpa*II sites were identified. A panel of primers was used to amplify overlapping gene segments of the promoter sequence. The PCR products from primer pair I contain two, primer pair III one, primer pair V one and primer pair VI one *Hpa*II sites. No differences in the results from restriction digest/PCR were observed among the eight MM cell lines, whether Sp17-positive or Sp17-negative, suggesting that the methylation status at these *Hpa*II sites may not be directly involved in the regulation of Sp17 gene expression (Figure 2

). The PCR products from primer pair II contain one *Hpa*II site. Using primer pair II, amplification products were only obtained from IM-9 and U266 cells. This indicates that this site is methylated in both cell lines and would be consistent with failure to express Sp17. However, using the same primer pair, no amplification products were obtained from the other two Sp17-negative cell lines, H929 and MM1-R, indicating that these *Hpa*II sites were hypomethylated in both cases. These findings therefore suggest that hypomethylation of the *Hpa*II site within primer pair II is not in itself sufficient to cause Sp17 gene expression. The PCR products from primer pair IV contain two *Hpa*II sites (positions −350 and −359). Using *Hpa*II restriction digest/PCR analysis, amplification products were obtained from all four Sp17-negative cell lines, H929, IM-9, MM1-R and U266, suggesting the methylation of either or both of the *Hpa*II sites within this gene segment of the promoter. In contrast, no PCR product was obtained from any of the four Sp17-positive tumour cell lines. The strong correlation between the results obtained from restriction digest/PCR and Sp17 status of the cell lines suggests that methylation at one or both of these *Hpa*II sites may be involved in the regulation of Sp17 gene expression.

### Confirmation of association between Sp17 gene expression and hypomethylation by bisulphite sequencing

Restriction digest/PCR analysis is sensitive to undigested DNA that will cause amplification of the unmethylated alleles. Therefore, to confirm the results obtained from restriction digest/PCR indicating differences in the methylation status of these *Hpa*II sites between Sp17-positive and Sp17-negative myeloma cells, genomic DNA from one Sp17-positive, KMS-11, and one Sp17-negative, IM-9, cells were selected for further analysis by bisulphite conversion. Following this, the genomic DNA underwent PCR and cloning. Eight randomly picked recombinant clones were subjected to nucleotide sequence analysis. We first examined the sequences at the eight *Hpa*II sites covered by the six primer pairs to validate the PCR primers we have used. A combination of methylated and demethylated sequences was obtained, indicating that the primers we have used were able to amplify both methylated and demethylated exon 1. Therefore, any results obtained were unlikely to be due to preferentially amplification of methylated sequences. We next examine the sequences to validate the results we have obtained in KMS-11 and IM-9 from restriction digest/PCR. A complete correlation was observed between the results from sequence analysis and those from restriction digest/PCR (Table 2

**Table 2:** % of methylated clones of Sp17+ and Sp17− myeloma cell lines

**Location within promoter**	**KMS-11 (Sp17+)**	**IM-9 (Sp17−)**	***P*-values**
*Hpa*II site IA	3/8 (37.5%)	6/8 (75%)	N.S.
*Hpa*II site IB	4/8 (50%)	6/8 (75%)	N.S.
*Hpa*II site II	0/8 (0%)	8/8 (100%)	*P*<0.001
*Hpa*II site III	3/8 (37.5%)	5/8 (62.5%)	N.S.
*Hpa*II site IVA	0/8 (0%)	8/8 (100%)	*P*<0.001
*Hpa*II site IVB	0/8 (0%)	7/8 (87.5%)	*P*<0.001
*Hpa*II site V	3/8 (37.5%)	5/8 (62.5%)	N.S.
*Hpa*II site VI	2/8 (25%)	6/8 (75%)	N.S.
Exon 1	82%	27%	*P*<0.001

). Only *Hpa*II sites within the gene segments from KMS-11 cells amplified by primer pairs II and IV showed complete demethylation, validating the results of the restriction digest/PCR that showed no PCR product after *Hpa*II digest. In contrast, the other *Hpa*II sites from KMS-11 or IM-9 all showed variable or complete methylation, as predicted by the restriction digest/PCR experiments. These results therefore support the notion that methylation at the two *Hpa*II sites covered by primer pair IV may be involved in the regulation of Sp17 gene expression. A total of 46 CpG sites were identified within the Sp17 exon 1. In addition to differences at the *Hpa*II sites, the 46 CpG sites were more hypomethylated in Sp17-positive KMS-11 cells (82%, range 80–98%) than Sp17-negative IM-9 cells (27%, range 4–50%) (*P*<0.001), indicating the association of Sp17 gene expression with global promoter hypomethylation (Figure 3

**Figure 3:**
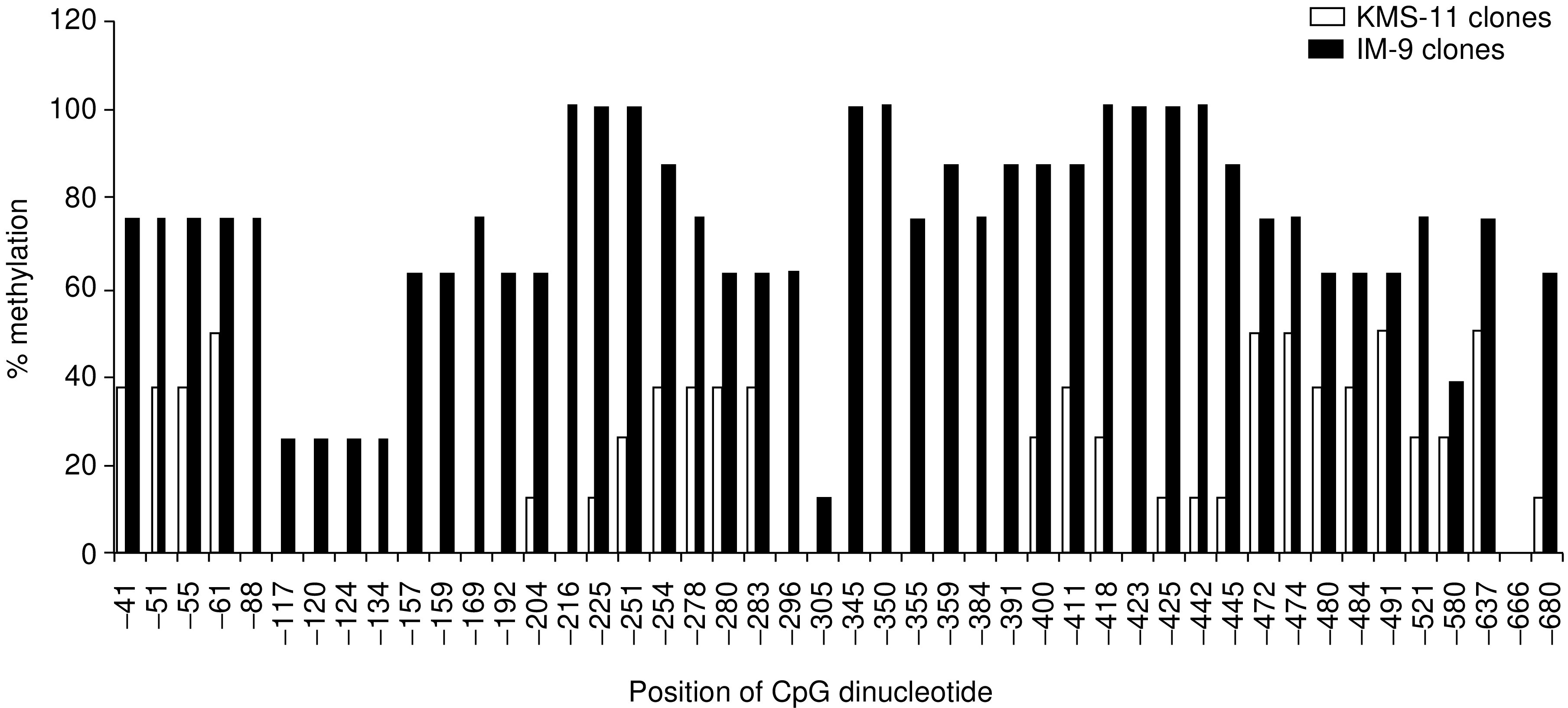
Comparison of the levels of methylation at all 46 CpG sites within exon 1 between KMS-11 and IM-9 cells, showing that there was increased hypomethylation throughout exon 1 in KMS-11 cells that express Sp17 gene.

).

### Suppression of CAT reporter gene transcription by *in vitro* methylation of Sp17 promoter

**Figure 4:**
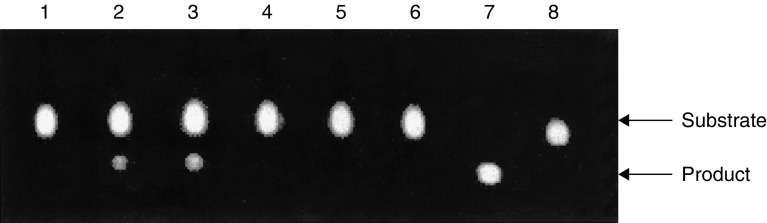
Analysis of 293 cells for CAT activities after plasmid transfection to confirm the role of Sp17 promoter methylation (lane 1=wild-type 293 cells; 293 cells transfected with pCAT^*^3 Enhancer-Sp17 promoter; lane 3=293 cells transfected with the positive control plasmid pCAT^*^3 Enhancer-SV40 promoter; lane 4=293 cells transfected with a control plasmid, pcDNA3.1; lane 5=293 cells transfected with pCAT^*^3 Enhancer plasmid that did not contain any promoter; lane 6=293 cells transfected with pCAT^*^3 Enhancer-Sp17 promoter/met; lane 7=product control; lane 8=substrate control).

Exon 1 of Sp17 gene is about 666 bp and is rich in CpG dinucleotides. It fits the criteria for being a CpG island that is often associated with a promoter region. To prove that exon 1 contains the Sp17 promoter sequence, we used the constructed pCAT^*^3 Enhancer-Sp17promoter in a transient transfection study on 293 human embryonic kidney cells. In contrast to cells transfected with the wild-type plasmid, CAT activity was obtained from lysate of cells transfected with pCAT^*^3 Enhancer-Sp17promoter, confirming that exon 1 contains Sp17 promoter sequence (Figure 4

).

To prove that methylation of the Sp17 promoter was sufficient to prevent expression, we constructed the reporter plasmids pCAT^*^3 Enhancer-Sp17promoter/met containing the Sp17 promoters upstream to the CAT report gene. Transient transfection of the plasmids into 293 human embryonic kidney cells with the unmethylated construct resulted in CAT gene expression; in contrast, no CAT activity was observed in transfectants containing the methylated plasmid (Figure 4). These results therefore confirm the role of Sp17 promoter methylation in the regulation of Sp17 gene expression.

### Upregulation of Sp17 gene expression in IM-9 by 5-azacytidine

**Figure 5:**
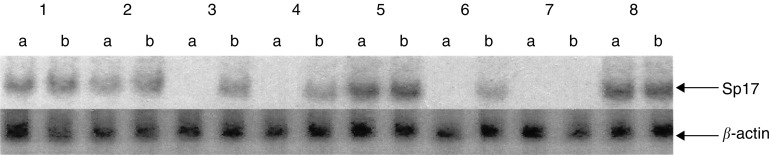
Northern blot analysis to compare the levels of Sp17 transcripts before (a) and after (b) exposure to 5-azacytidine (lane 1=ARK-B; lane 2=ARP-1; lane 3=H929; lane 4=IM-9; lane 5=KMS-11; lane 6=MM1-R; lane 7=U266; and lane 8=RPMI 8226).

To determine if DNA hypomethylating agents could upregulate the expression of Sp17 gene, we exposed all eight tumour cell lines to 5-azacytidine (2 *μ*M) for 96 h. Following culture in control or 5-azacytidine-containing mediums, total RNA from these cells was isolated for Northern blot analysis. Autoradiographic signals corresponding to Sp17 transcripts were only obtained in the four Sp17-positive tumour cells (ARK-B, ARP-1, KMS-11 and RPMI 8226) when the tumour cells were cultured in medium without any 5-azacytidine (Figure 5

). In keeping with the results suggested by RT-PCR, the Sp17 signal was strongest in KMS-11 and weakest in ARP-1. No Sp17 signal was observed with H929, IM-9, MM1-R and U266 cells. However, following culture in 5-azacytidine, Sp17 transcript levels were further increased in ARK-B and ARP-1 cells. In addition, the 5-azacytidine also induced the expression of Sp17 gene in H929, IM-9 and MM1-R cells that originally did not express Sp17. When each tumour cell line in 5-azacytidine was compared to that of its respective control culture, no differences were observed in the viability or rate of proliferation of the cells. The increase in the levels of Sp17 transcripts following exposure to 5-azacytidine was therefore due to the upregulation of gene expression rather than selection of Sp17-positive variants within individual cell lines. To prove that the upregulation of Sp17 gene expression was related to promoter hypomethylation, bisulphite conversion was carried out using genomic DNA from IM-9 cells, before and after preincubation in 5-azacytidine. Significantly more clones showed hypomethylation at the two *Hpa*II sites amplified by primer pair IV (at position −350, increased from 0 to 62.5% and at position −359; increased from 12.5 to 50%; *P*<0.001) after exposure to 5-azacytidine, further supporting the possible involvement of the sequence around these two sites in the control of Sp17 gene expression. Compared to IM-9 cells that did not express Sp17 gene when cultured in control medium, the promoter sequence of IM-9 cells preincubated with 5-azacytidine also showed more global hypomethylation at the CpG islands (69 *vs* 27%, *P*<0.0005).

## DISCUSSION

CT antigens are potentially suitable targets for cancer immunotherapy because of their limited expression in normal tissues and their *in vivo* immunogenicity (Reynolds *et al*, 1997; Stockert *et al*, 1998; Traversari, 1999). Since these targets are often not essential for the neoplastic process or survival of the tumour cells, it is not uncommon to observe heterogeneity of expression of these targets within a tumour lesion. Identification of the mechanisms regulating the expression of the target genes may therefore lead to new approaches that upregulate gene expression and circumvent deficiency within individual patients to improve the outcome of immunotherapy.

Genetic anomalies occur frequently in MM. These anomalies, resulted from increased CpG island and decrease global DNA methylation, lead to the inactivation of many well-characterised tumour suppressor genes as well as the inactivation of DNA repair genes. It has been proposed that the expression of genes with CpG-rich promoters is normally controlled via tissue-specific transcription factors (Eden and Cedar, 1994), while for those with CpG-poor promoters, gene expression is controlled through DNA methylation (Bird, 1992). However, exceptions can occur. Recent studies have indicated that DNA methylation, instead of tissue-specific transcription factors, is the primary regulatory mechanisms for the control of expression on a subset of male CpG-rich germline-specific genes, the *MAGE*-type genes (De Smet *et al*, 1999).

Unlike the genes for most other CT antigens, the gene encoding Sp17 is localised to chromosome 11q rather than X chromosome. Nevertheless, Sp17 gene expression also exhibits a very restricted normal tissue expression pattern identical to those by genes encoding some CT antigens (Lim *et al*, 2001; De Jong *et al*, 2002), suggesting its potential as a target for immunotherapy of MM and other tumours. Its possible applicability in tumour vaccines has been supported by *in vitro* data showing the ability to generate tumour-specific CTLs from these patients (Chiriva-Internati *et al*, 2002a, 2002c). However, its applicability may be limited since Sp17 was only detected in 27% of MM patients. In the light of the strong therapeutic and diagnostic implications of Sp17 expression, we have set out to determine the role of DNA methylation in regulating the presence and levels of expression of Sp17 in MM.

In keeping with our previous results involving the study of fresh MM tumour cells (Lim *et al*, 2001), we first showed that not all MM tumour cell lines express Sp17. The heterogeneity of expression of Sp17 in MM suggests the lack of functional necessity of Sp17 gene products in maintaining the malignant phenotypes. Analysis of the 5′ region in exon 1 of the gene showed that it is CpG rich, suggesting the possible promoter function of the sequence. The promoter function of exon 1 was further confirmed by *in vitro* transfection studies showing the ability of exon 1 sequence to allow the transcription and expression of CAT gene in 293 cells. Using methylation-sensitive restriction digest/PCR analysis in screening studies, the CpG region most susceptible to the inactivating action of methylation was identified by mapping of the different overlapping gene segments amplified from exon 1. Differences in the degree of methylation of the CpG sites within the Sp17 promoter sequence were compared and confirmed by bisulphite conversion and correlated to Sp17 expression in these tumour cell lines. Analysis of the sequences of the clones indicates some heterogeneity of DNA methylation within a tumour cell line. The role of promoter methylation in silencing the Sp17 gene in Sp17-negative tumour cells was confirmed by demonstrating the suppression of the CAT gene expression by *in vitro* methylation of exon 1. These results therefore provide the first evidence that the expression of Sp17 gene in MM cell lines is directly dependent on the methylation status of distinct CpG dinucleotides located in the promoter regions, a scenario not unlike that in *MAGE* genes (De Smet *et al*, 1999).

*In vitro* exposure of these MM cells to the DNA hypomethylating agent, 5-azacytidine, has been recently shown to induce the expression of different CT antigens (Weiser *et al*, 2001; Sigalotti *et al*, 2002). In the present study, we have found that Sp17 gene could also be upregulated by exposure of the tumour cells to 5-azacytidine, except in U266. This finding, nevertheless, further provides evidence that DNA methylation is involved in the expression of Sp17 by MM cells. The reason for the failure to upregulate Sp17 expression in U266 is unclear and remains speculative. Differences in the metabolism of 5-azacytidine by different myeloma cell lines may explain this. It should, however, be noted that the present study does not support the use of 5-azacytidine to increase the expression of Sp17 *in vivo*, since the concentration of the hypomethylating agent used in our experiments was much higher than the tolerable dose in human (Weiser *et al*, 2001). Whether or not other hypomethylating agents, when used at therapeutic doses, will promote the expression of Sp17 remains to be determined.

Various workers have previously demonstrated that changes in the methylation of the genomes frequently occur in MM (Mays-Hoopes *et al*, 1986; Ng *et al*, 1997). Our results, therefore, suggest that Sp17 is the expression in myeloma cells as the consequence of promoter demethylation. Since changes in gene methylation become more frequent as the disease progresses, it is expected that the expression of Sp17 in myeloma cells will increase as MM becomes more advanced.

We previously found that mRNA encoding Sp17 was detected only in the testis and not any other normal tissues (Lim *et al*, 2001). Our findings are in keeping with previous investigations of Sp17 expression using Northern blot analysis in rabbit and baboon (Richardson *et al*, 1994; Adoyo *et al*, 1997). Using RT–PCR, De Jong *et al* (2002) were also unable to detect Sp17 mRNA in any normal tissues except the testis. Weak bands of PCR products were, however, obtained in several normal tissues, but only after two rounds of 30-cycle PCR, suggesting that very low level of Sp17 transcripts are present in some normal tissues. However, a recent study by Frayne and Hall (2002) produced contrasting results. They demonstrated that Sp17 transcripts could be detected, albeit at much lower levels than the testis, in many normal tissues after only one 30-cycle PCR. The apparent discrepancies of results suggest that it is likely Sp17 is expressed at low levels in some normal tissues. Other CT antigens such as NY-ESO-1, CT15/fertilin *β* and CT-16 are undetectable at 35 cycles of conventional RT–PCR but have been detected in a limited number of normal tissues at 40 cycles of real-time quantitative RT–PCR (Scanlan *et al*, 2002). However, the levels were less than 3% of the level detected in normal testis and the biological meaning of such low-level expression is unclear. In the light of the results from the present study, the apparent conflicting data are not surprising because random DNA methylation/demethylation errors can occur and are responsible for leaking expression of the Sp17 genes in some normal tissues.

In conclusion, we have elucidated the mechanisms responsible for the regulation of Sp17 gene expression in MM tumour cell lines. Although DNA methylation is not the primary control mechanism regulating the expression of most tissue-specific genes, our results indicate that promoter methylation can serve as the main regulatory mechanism for the expression of Sp17 in tumour cell lines. The susceptibility to induction of Sp17 in tumour cells by hypomethylating agent further supports the role of DNA methylation in silencing Sp17 gene. Our findings may have therapeutic implications and provides possible explanation to the leaky expression of Sp17 in some normal tissues observed in some studies.
